# Variation in Indole-3-Acetic Acid Production by Wild *Saccharomyces cerevisiae* and *S*. *paradoxus* Strains from Diverse Ecological Sources and Its Effect on Growth

**DOI:** 10.1371/journal.pone.0160524

**Published:** 2016-08-02

**Authors:** Yen-Yu Liu, Hung-Wei Chen, Jui-Yu Chou

**Affiliations:** Department of Biology, National Changhua University of Education, Changhua 500, Taiwan, R.O.C; Tulane University Health Sciences Center, UNITED STATES

## Abstract

Phytohormone indole-3-acetic acid (IAA) is the most common naturally occurring and most thoroughly studied plant growth regulator. Microbial synthesis of IAA has long been known. Microbial IAA biosynthesis has been proposed as possibly occurring through multiple pathways, as has been proven in plants. However, the biosynthetic pathways of IAA and the ecological roles of IAA in yeast have not been widely studied. In this study, we investigated the variation in IAA production and its effect on the growth of *Saccharomyces cerevisiae* and its closest relative *Saccharomyces paradoxus* yeasts from diverse ecological sources. We found that almost all *Saccharomyces* yeasts produced IAA when cultured in medium supplemented with the primary precursor of IAA, L-tryptophan (L-Trp). However, when cultured in medium without L-Trp, IAA production was only detected in three strains. Furthermore, exogenous added IAA exerted stimulatory and inhibitory effects on yeast growth. Interestingly, a negative correlation was observed between the amount of IAA production in the yeast cultures and the IAA inhibition ratio of their growth.

## Introduction

Plants and microorganisms, including bacteria, yeasts, and multicellular fungi, can produce indole-3-acetic acid (IAA) [1–4]. IAA is the most common plant hormone and is classified as an indole derivative of the auxin family. IAA plays several vital roles in regulating various developmental and physiological processes in plants; it also increases plant protection from external stress [1, 5, 6]. Thus, studies are under way globally to exploit the potential for developing IAA-producing microbes to promote plant growth and protect plants for sustainable agriculture [7]. However, the roles of microbial IAA in species interaction networks have received only limited attention. It has already been shown that IAA regulates cell differentiation and affects gene expression in several microorganisms; this finding supports the role of IAA as a signaling molecule that regulates microbial growth [8–11]. IAA biosynthesis pathways have been broadly studied in several groups of bacteria [12, 13]. However, few studies have investigated the IAA biosynthesis pathway in yeast [11, 14].

*Saccharomyces* is a genus of fungi that includes many species of yeasts, and many members of this genus are considered valuable to food production. The most valuable species is *Saccharomyces cerevisiae*, which has been used in brewing and bakery processes for thousands of years [15]. Thus, this has led to the idea that *S*. *cerevisiae* is a domesticated species. It is also an attractive model organism because its genome has been sequenced; it replicates rapidly and is easy to manipulate genetically; and it is easy to maintain and manipulate in the laboratory. *S*. *cerevisiae* and *Saccharomyces paradoxus* are known to coexist in the same location in the wild but display different levels of reproductive isolation [16–18]. Although *S*. *paradoxus* coexists globally with *S*. *cerevisiae*, it is not associated with humans. *S*. *paradoxus* is commonly found on exudates and bark of deciduous trees and in associated soils [16, 17]. Thus, it has been proposed as an improved model for ecology, population genomics, and phylogenetic studies, and the characteristics of this wild species has been compared with those of laboratory yeasts [19, 20]. However, the ecologically relevant trait variation of natural populations of yeasts has rarely been systematically investigated [21, 22]. Liti et al. [23] collected *S*. *cerevisiae* and *S*. *paradoxus* from a large variety of sources and locations, including pathogenic, baking, wine, food spoilage, natural fermentation, sake, probiotic, and plant isolates. This collection of yeast isolates for the Saccharomyces Genome Resequencing Project enables the examination of valuable phenotypes that exist in natural yeasts but are not prevalent in laboratory strains [24, 25]. This provides an extended resource for examining phenotypic variation (ex: IAA production ability) in natural isolates. Thus, this study aims to assess the variation in IAA production and its effect on the growth of wild *S*. *cerevisiae* and *S*. *paradoxus* strains from diverse ecological sources.

## Materials and Methods

### Yeast strains

The *S*. *cerevisiae* and *S*. *paradoxus* strains from various sources and locations used in this study were gifts from Dr. Gianni Liti (Institute for Research on Cancer and Ageing of Nice, University of Nice Sophia Antipolis, France) and Dr. Jun-Yi Leu (Institute of Molecular Biology, Academia Sinica, Taiwan). The detailed information can be found in S1 Table in this study and Table S1–S2 in the study of Liti et al. [23].

### Quantification of IAA by using Salkowski colorimetric assay

To quantify the IAA produced, yeast isolates were grown in a test tube in yeast extract–peptone–dextrose (YPD; 1% yeast extract, 2% peptone, 2% dextrose) medium with or without 0.1% (w/v) L-tryptophan (L-Trp) and incubated in the dark on a shaker at 30°C and 150 rpm. Thereafter, 1 mL of the cells were pelleted by centrifugation at 3000*g* for 5 min, and 0.5 mL of the supernatant was mixed with 0.5 mL of the Salkowski reagent (2 mL of 0.5M iron(III) chloride and 98 mL of 35% perchloric acid) [26]. After 30 min, color development (red) was quantified using a spectrophotometer (Unico 1200-Spectrophotometer, USA) at 530 nm. A calibration curve using pure IAA was established to calculate the IAA concentration.

### Effects of exogenous IAA on yeast growth

To determine the possible biological role of IAA in yeast, the effects of exogenous IAA on the growth of the test yeasts were investigated by adding 5 mM chemically synthesized IAA (Sigma-Aldrich, USA) to YPD medium. The growth of yeast under IAA treatment was monitored by measuring the optical density at 660 nm with a spectrophotometer. The cultures were vigorous stirred/shaken before the O.D. measurement. The strains which the flocculation was strong and irreversible were not included in our study. The YPD medium without exogenous IAA served as the control. The inhibition ratio (%) was calculated using the following formula: inhibition ratio (%) = [(A − B)/A] × 100, where A is the average generation time of log-phase cells of yeast under IAA treatment (5 mM), and B is the average generation time of log-phase cells of the control group. Data are expressed as mean ± standard deviation (SD). The significance of differences between the experimental and control groups was determined using the Student t test and analysis of variance (ANOVA). P < 0.05 was considered statistically significant. ANOVA was used to determine the differences between the experimental groups, and the Tukey–Kramer method was used in ANOVA to create confidence intervals for all pairwise differences.

### Coculture competition assays

The cells of the test (strain UWOPS05-217.3) and reference (strain W303 with the fluorescently labeled expressing the Pgk-YFP fusion protein) strains were adapted to media for 24 h. Subsequently, the test strains were refreshed and grown to the mid-log phase (0.4 < O.D. < 1) and mixed in a 1:1 ratio with the reference strain. These counts provided the initial ratios of the two strains for competition. Yeast growth was monitored until 30 h after treatment. The ratio of the two competitors was quantified at the initial and final time points under an upright fluorescent microscope (Leica DM2500). Three replicates of this experiment were performed to measure variations.

## Results

### Profile of IAA production by yeasts grown in YPD medium with 0.1% (w/v) L-Trp

**Fig 1:**
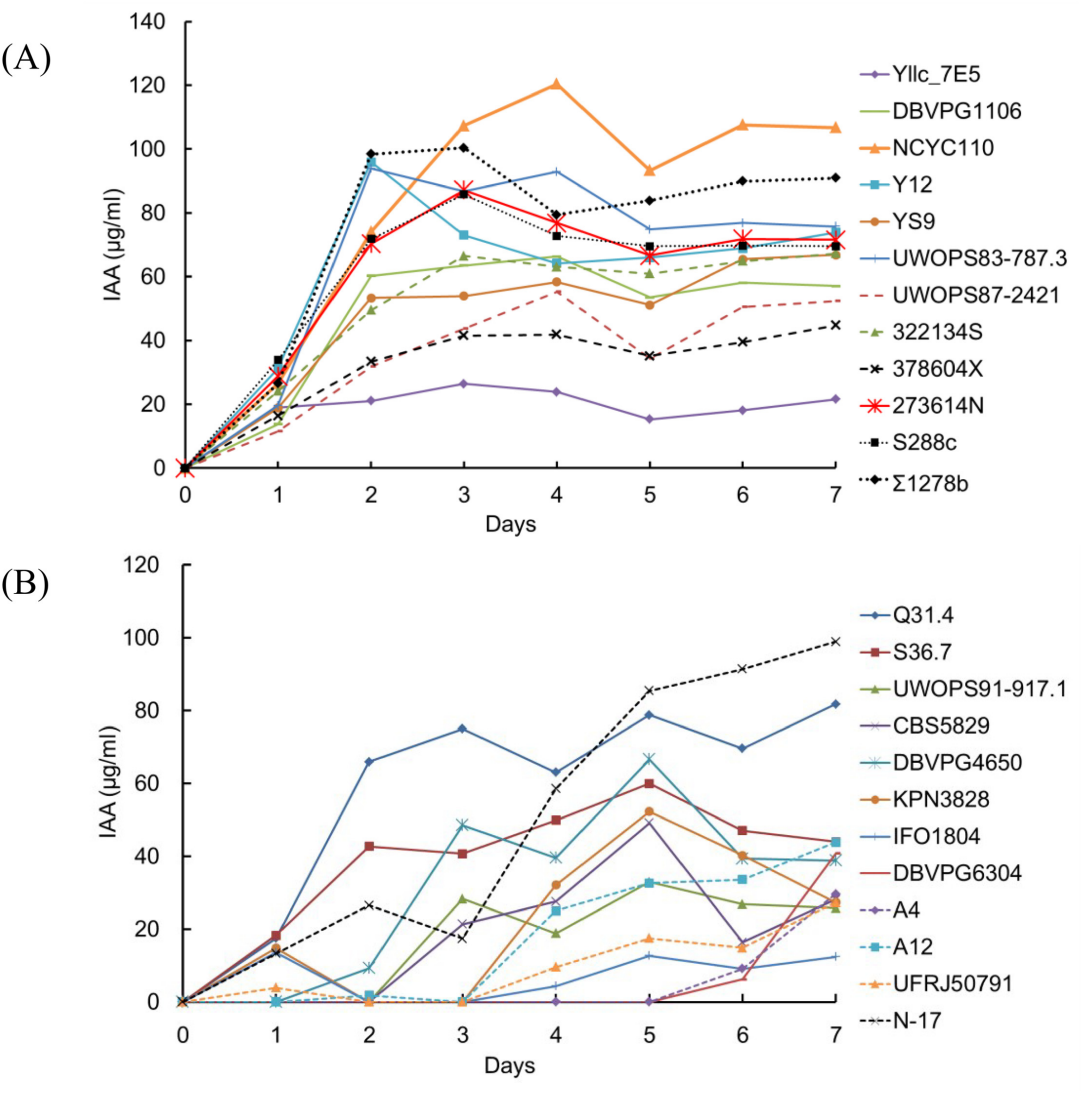
Profile of IAA production by yeasts in YPD medium with 0.1% (w/v) L-tryptophan. IAA production by yeasts was studied for 1 week, and maximum production was observed on day 3 in most *S*. *cerevisiae* strains (A) and on day 5 in most *S*. *paradoxus* strains (B). Only the mean of the three replicates of each strain was shown.

To examine the profile of IAA production by yeasts, different strains from each clade of neighbor-joining phylogenetic trees, on the basis on pairwise SNP differences in the study of Liti et al. [23], were used. Fig 1 depicts the time course of IAA production by the wild *S*. *cerevisiae* and *S*. *paradoxus* strains grown in YPD medium containing 0.1% (w/v) L-Trp. IAA production by these wild yeasts over 1 week was studied; maximum production was observed on day 3 for most *S*. *cerevisiae* strains (Fig 1A) and on day 5 for most *S*. *paradoxus* strains (Fig 1B). However, maximum production by the *S*. *paradoxus* strains DBVPG6304 and A4 started to accumulate IAA until day 5. Thus, maximum IAA production by all wild *S*. *cerevisiae* and *S*. *paradoxus* strains was recorded on days 3 and 5, respectively, in the next experiment.

### Natural isolates exhibit considerable diversity in IAA production

**Fig 2:**
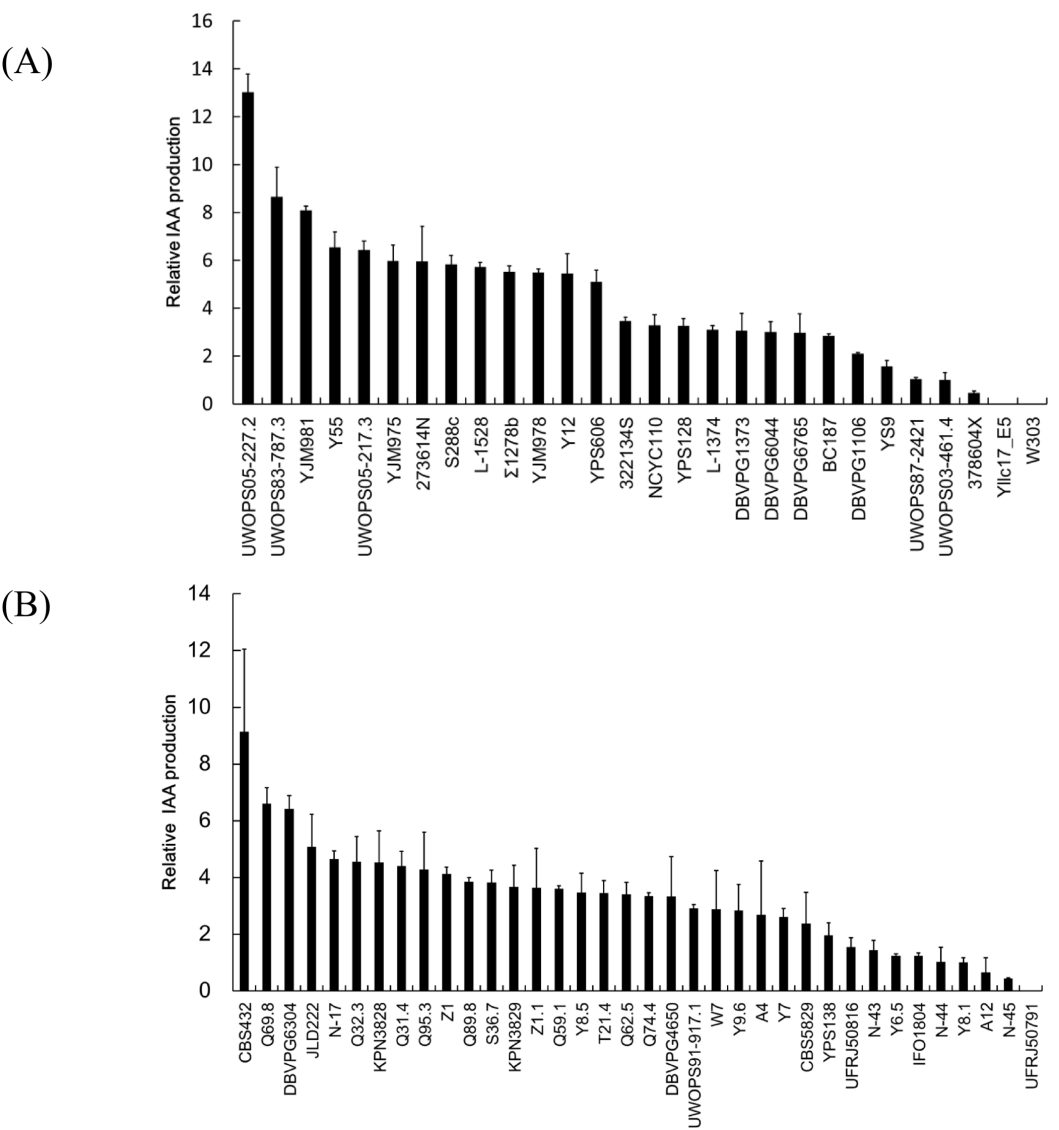
Natural *Saccharomyces* yeast isolates exhibit considerable diversity in IAA production. The value of IAA production represented in this Fig is derived by the amount of IAA produced by each strain relative to that produced by strain UWOPS03-461.4 for *S*. *cerevisiae* (A) and relative to that produced by strain Y8.1 for *S*. *paradoxus* (B).

To determine the diversity of IAA production present in the collection of strains from the Saccharomyces Genome Resequencing Project, we examined 28 *S*. *cerevisiae* strains and 36 *S*. *paradoxus* strains from the collection and a laboratory strain control (strain W303). The value of IAA production represented in Fig 2 is derived by the amount of IAA produced by each strain relative to that produced by strain UWOPS03-461.4 for *S*. *cerevisiae* and relative to that produced by strain Y8.1 for *S*. *paradoxus*. IAA production ranged from 287.0 (± 16.6) (strain UWOPS05-227.2) to 14.5 (± 2.5) μg/mL (strain 378604X) for *S*. *cerevisiae* and from 238.4 (± 76.2) (strain CBS432) to 11.3 (± 1.0) μg/mL (strain N-45) for *S*. *paradoxus* (S1 Table). However, IAA production by the *S*. *cerevisiae* strains Yllc17_E5 and W303 and the *S*. *paradoxus* strain UFRJ50791 was not detected using the colorimetric method in this study.

IAA was the first plant hormone discovered, but its biosynthetic pathway remains unclear at the genetic level. To confirm the existence of a Trp-independent pathway in *Saccharomyces* yeasts, we analyzed IAA production by yeast cultured in medium without Trp. Because the working range of a spectrophotometer is usually 0.1–1.0. According to the calibration curve using pure IAA we established for calculating IAA concentration, O.D. 0.1 is around 20 μg/mL. Thus, the IAA produced by yeasts more than 20 μg/mL will be considered as IAA producing yeasts. We observed that among all evaluated yeast isolates, only three strains produced IAA in the absence of exogenous Trp. For *S*. *cerevisiae*, only strains UWOPS05-227.2 (100.24 ± 6.33 μg/mL) and UWOPS05-217.3 (35.8 ± 3.3 μg/mL) produced IAA in the absence of exogenous Trp (S1 Table). Strain UWOPS05-227.2 was collected from a stingless bee (*Trigona* spp.) near a Bertam palm flower, and strain UWOPS05-217.3 was collected from the nectar of Bertram palm. Both strains were collected in Telok Senangin, Malaysia. For *S*. *paradoxus*, only strain Q31.4 (52.1 ± 6.6 μg/mL) produced IAA in the absence of exogenous Trp (S1 Table). Strain Q31.4 was collected from the bark of *Quercus* spp. in Windsor Great Park, UK. These three strains can produce IAA in YPD medium without exogenous Trp; however, the amount of IAA produced is lower compared with that produced in YPD medium supplemented with Trp.

### Effects of exogenous IAA on yeast growth

**Fig 3:**
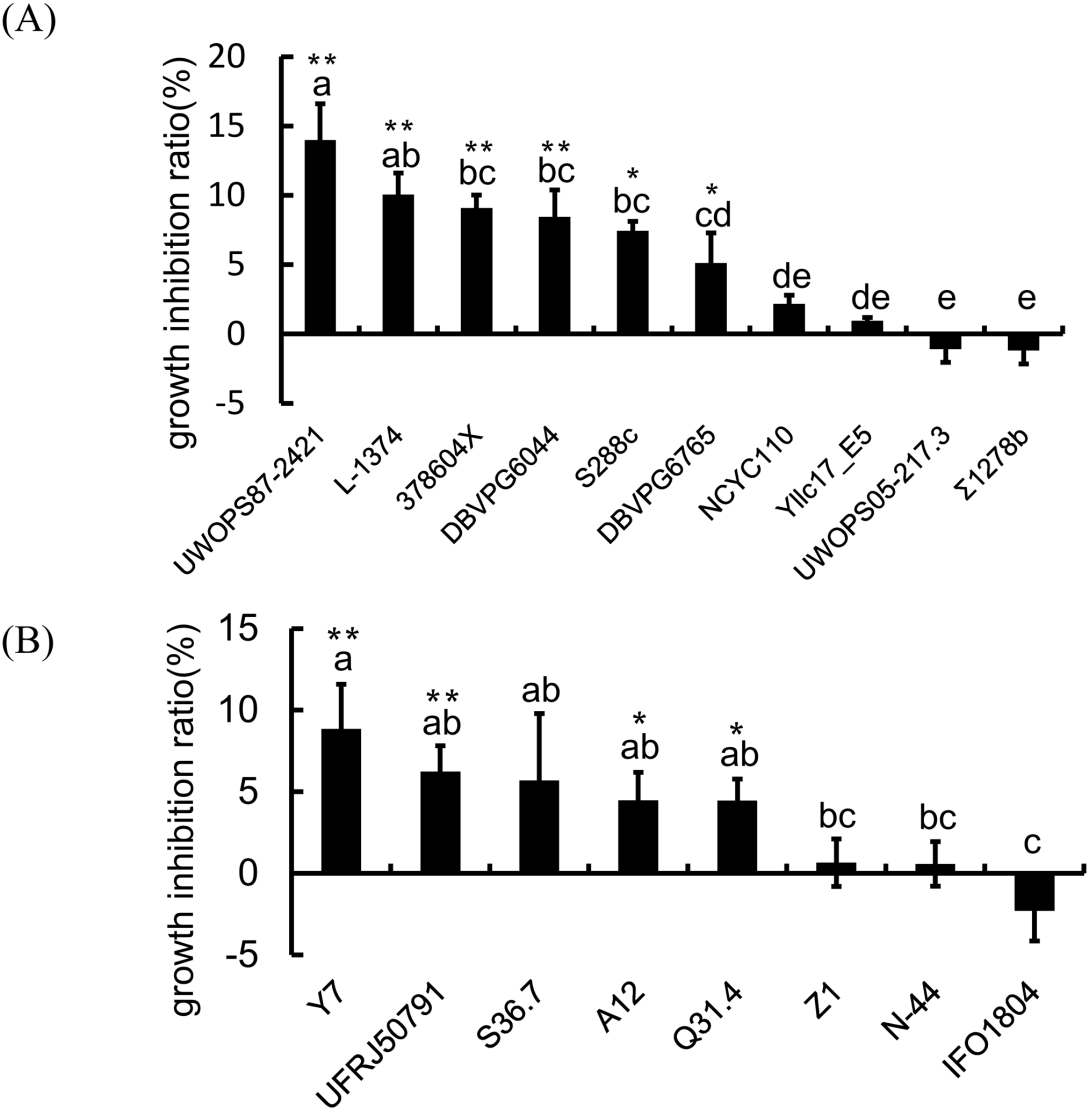
Effects of exogenous IAA on yeast growth. Yeasts were grown in YPD medium containing 5 mM IAA. Yeasts grown in YPD medium without exogenous IAA served as control. The inhibition ratio (%) was calculated using the following formula: inhibition ratio (%) = [(A − B)/B] × 100, where A is the average generation time of log-phase cells of yeast under IAA treatment (5 mM), and B is the average generation time of log-phase cells of the control group. (A) For *S*. *cerevisiae*, the high IAA concentrations substantially reduced the growth of strains UWOPS87-2421, L-1374, 378604X, DBVPG6044, S288C, and DBVPG6765, but exerted no effects on strains NCYC110 and Yllc17_E5. IAA treatment slightly promoted the growth of strain UWOPS05-217.3 and ∑1278b, although not significant. (B) For *S*. *paradoxus*, the high IAA concentrations substantially reduced the growth of strains Q31.4, Y7, and UFRJ50791, but exerted no effects on strains Z1 and N-44. IAA treatment slightly promoted the growth of strain IFO1804, but not significant.

**Fig 4:**
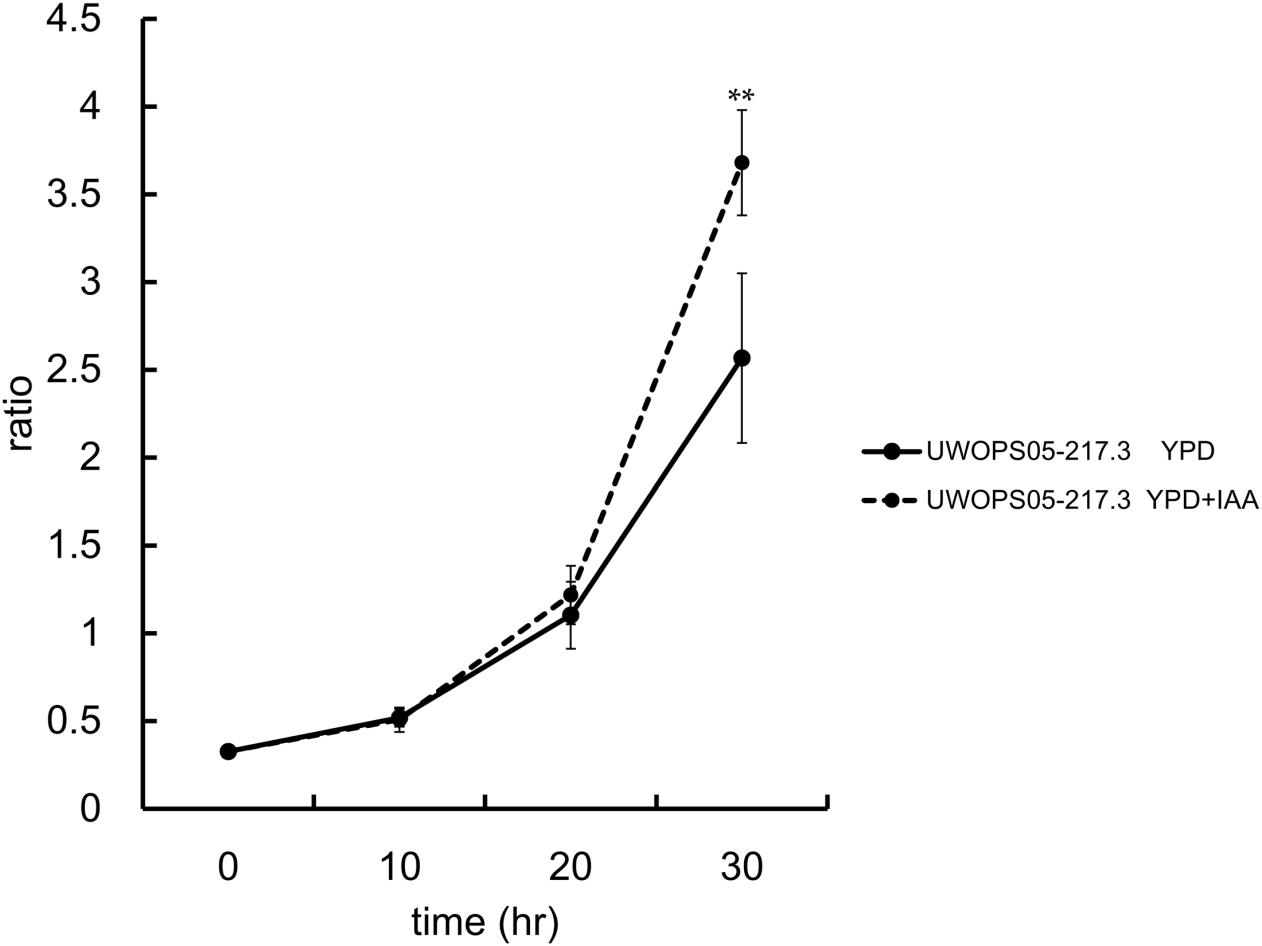
Result of coculture competition assays. The counts showed the initial ratio and the final ratio between the IAA-tolerant strain *S*. *cerevisiae* UWOPS05-217.3 to the IAA-sensative reference strain. Thirty hours after treatment, the counts indicated the final ratio in YPD medium with exogenous IAA was significantly higher than the ratio in YPD medium without exogenous IAA. The difference in the final ratio between these two treatments was significant (P < 0.01).

Our previous study indicated that low exogenous IAA concentrations promote the growth of some plant-associated yeast species; however, high IAA concentrations (5 mM IAA) inhibit the growth of these yeasts [27]. In the current study, we found IAA exerts stimulatory and inhibitory effects at the intra-species level. In Fig 3, it presents the data of some representative strains. *S*. *cerevisiae* strains S288c and ∑1278b are two of the most widely used laboratory strains and served as controls to compare the differences in the wild strains. For *S*. *cerevisiae*, the results showed that the high IAA concentrations substantially reduced the growth of strains UWOPS87-2421, L-1374, 378604X, DBVPG6044, S288C, and DBVPG6765 but exerted no effects on strains NCYC110 and Yllc17_E5. IAA treatment slightly promoted the growth of strain UWOPS05-217.3 and ∑1278b, though this effect was not significant (Fig 3A). For *S*. *paradoxus*, the results showed that the high IAA concentrations substantially reduced the growth of strains Y7, UFRJ50791, A12, and Q31.4 but exerted no effects on strains Z1, N-44, and S36.7. Similar to the finding obtained for the strain *S*. *cerevisiae* UWOPS05-217.3, IAA treatment slightly promoted the growth of strain IFO1804, though this result did not reach significance (Fig 3B). Furthermore, to investigate the role of environmental IAA in intra-specific competition, we used coculture competition assays between the test *S*. *cerevisiae* strain UWOPS05-217.3 and the reference strain (strain W303 with the fluorescently labeled). Thirty hours after treatment, the counts indicated that the final ratio of *S*. *cerevisiae* UWOPS05-217.3 to the reference strain in YPD medium with exogenous IAA was significantly higher than the ratio in YPD medium without exogenous IAA (Fig 4). A significant difference was observed in the final ratio between these two treatments (P < 0.01). These results suggest that the *S*. *cerevisiae* strain UWOPS05-217.3 is more favorably adapted to an environment with a high IAA concentration than the sensitive strains are.

### Relationship between IAA production and influence of exogenous IAA on growth

**Fig 5:**
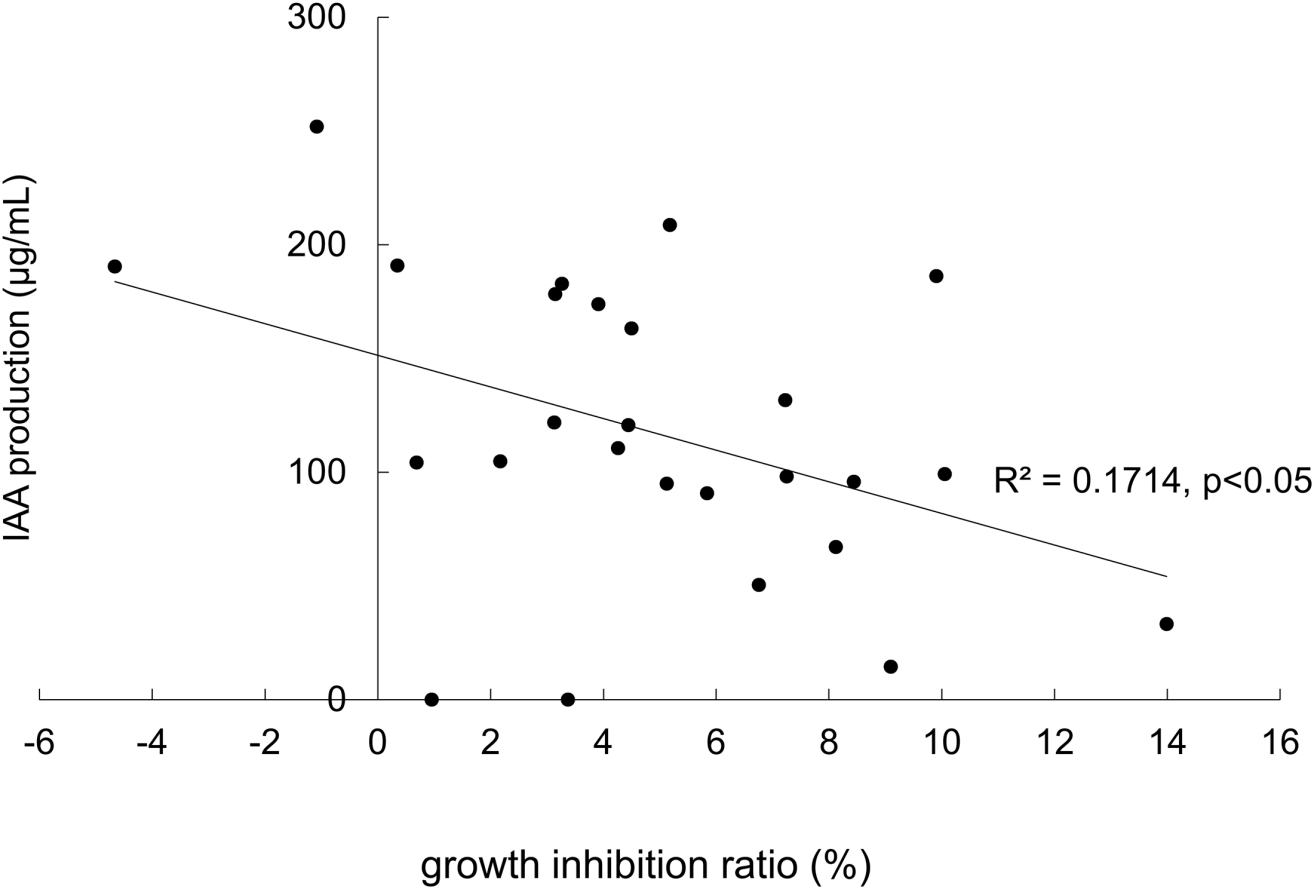
A negative correlation was observed between the amount of IAA production in the yeast cultures with Trp and the IAA inhibition ratio of the growth of yeasts. Scatter plot of the Pearson’s correlation analysis between IAA production (μg/mL) and the growth inhibition ratio (%) from 26 strains of *S*. *cerevisiae*. (Pearson’s correlation coefficient r = −0.414, P < 0.05).

To investigate the correlation between IAA production by yeasts and the influence of exogenous IAA on their growth, we measured the IAA inhibition ratio of the growth rate of each strain. As shown in Fig 5, a negative correlation was observed between IAA production in the cultures with Trp for each strain on the y axis and the inhibition ratio plotted on the x axis (Pearson’s r = −0.414; P < 0.05). The results were obtained from 26 *S*. *cerevisiae* strains. IAA treatment slightly promoted the growth of two strains, which produced relatively high amounts of IAA.

## Discussion

Tryptophan was proposed as a precursor of IAA in fungi, which was confirmed in later studies not only in certain fungi but also in *Saccharomyces* yeasts [11, 28–30]. Tryptophan may be expensive in terms of the energy cost to the microorganism because of its complex double ring structure and the high energy requirement for its synthesis [31]. Thus, tryptophan is not biosynthesized by all microorganisms, and these microorganisms rely on their plant hosts or surrounding microbial sources [32]. Moreover, the ability to convert tryptophan to IAA by these microorganisms (“guests”) can also benefit the tryptophan provider (“host”), which may be seen as an example of mutualism [27, 33]. However, tryptophan may not always be available or adequate for the microbes to synthesize IAA in natural environments. A Trp-independent pathway for IAA synthesis has been proposed to exist in several bacteria and yeast species [1, 11]. IAA generated through the Trp-independent pathway is proposed to branch from either indole or indole-3-glycerol phosphate, both precursors of tryptophan; however, no specific enzymes or genes of this pathway have been identified yet [2, 11, 29, 34]. In addition to tryptophan-dependent pathways, a tryptophan-independent pathway has been identified in the free-living soil bacterium *Azospirillum brasilense* [35]. Thus, *A*. *brasilense* is a well-known plant growth-promoting rhizobacteria (PGPR) that can positively influence plant growth because it produces a substantial amount of IAA more easily.

Rao et al. [11] used a mutant *S*. *cerevisiae* strain with deletions in two aldehyde dehydrogenase (*ALD*) genes hypothesized to catalyze the ultimate step in IAA production. They found that the mutant strain could not convert radiolabeled Trp to IAA but could produce IAA in the absence of exogenous Trp, and that the strain could even produce higher IAA concentrations than the wild-type strain. This result suggests that the *S*. *cerevisiae* strain may have evolved multiple pathways for IAA synthesis, one of which is Trp-independent. However, Xin et al. [36] found that no detectable IAA was produced by a *S*. *cerevisiae* strain (Lesaffre yeast corporation, Milwaukee, WI) after 7-day incubation with or without L-Trp. In this study, we found that only three *Saccharomyces* yeast strains produced IAA in medium without L-Trp. These data suggest that the Trp-independent pathway for IAA biosynthesis independently evolved multiple times in *Saccharomyces* yeasts. From the neighbour-joining phylogenetic trees based on pairwise SNP differences in Liti et al. [23], the *Saccharomyces* yeasts produced IAA when cultured in medium supplemented without the L-Trp fall into the different clades. Thus, it suggests that the Trp independent pathway for IAA biosynthesis has independently evolved multiple times during *Saccharomyces* yeast evolutionary history. But one thing to notice is that Salkowski's reagent test we used in this study is a primary screening for indolic substances. It is useful, though, for screening large yeast populations, as it is fast, thus reducing sample size, time and cost. However, the IAA production in these yeasts should be further confirmed by high-performance liquid chromatography (HPLC) or gas chromatography-mass spectrophotometer (GC- MS) analysis.

From genome expression profiling of yeast cells (strain Σ1278b) treated with IAA, Prusty et al. [10] identified Yap1, a basic leucine zipper transcription factor, as a key mediator of this response. They further indicated that the wild type strain Σ1278b is sensitive to IAA and the *yap1-1* mutant is hypersensitive to IAA effects on growth inhibition because they accumulate more IAA than can wild type. However, strain ∑1278 in our analysis is not sensitive to IAA. In their study, the OD at 600 nm was measured after 2 days of incubation (stationary phase). However, we measured the average generation time of log-phase cells in this study. It may causes the differences between these two studies. Even though, it will be interesting to compare the sequences of wild yeast strains used here to identify any mutations or sequence variability related to Yap1 among them that show differences in IAA sensitivity. However, by both nucleotide and amino acid sequence alignments, *YAP1* shows high sequence similarity of the yeast strains we used in IAA sensitivity experiment (Fig 3). The mutations among them are not related to the level of IAA sensitivity according to the results in Fig 3. It needs further investigation on the connection between Yap1 variation and IAA sensitivity in yeasts.

Microbes have evolved means of interference competition to gain an advantage over their ecological competitors. Interference competition is a direct form of competition in which an organism actively interferes with the ability of another organism to obtain its resource. In this situation, they can evolve behavioral strategies and morphologies to outcompete rivals in their population. The use of secreted antagonistic compounds by yeast cells is one such prominent example [37, 38]. In our previous study [27], low concentrations of exogenous IAA did not influence or even promote yeast growth; however, high IAA concentrations substantially reduced yeast growth. Previous studies have similarly indicated that IAA inhibits the growth of fungi [10, 39]. Thus, we conclude that IAA can exert stimulatory and inhibitory effects on fungal growth. However, in this study, a high amount of IAA (5 mM) was used to assay the effects of exogenous IAA on yeast growth. Some concerns exist that this concentration does not reflect all actual concentrations found in the wild or those produced by microbes. In this study, the highest IAA concentration secreted by yeast was approximately 287.0 μg/mL (strain UWOPS05-227.2), which is equal to 1.6 mM. According to Nutaratat et al. [40], the red yeast *Rhodosporidium paludigenum* even can secrete 1623.9 μg/mL IAA (approximately 9 mM). Furthermore, the IAA measured in this study was secreted by yeast into liquid medium. We believe that the amount of IAA produced by yeasts in the microniche under wild conditions is higher than that measured in this study. Furthermore, we used a coculture assay to assess the influence of IAA on the growth rate of the IAA-sensitive reference strain and IAA-tolerant test strains. In this study, the IAA-sensitive reference strain expressed the Pgk-YFP fusion protein. Using this method, we clearly determined the effect of IAA on the growth rate.

Another well-established antagonistic behavior of yeast is the production of yeast killer toxins [41, 42]. The killer activity of yeast is readily detectable only when a suitable sensitive strain is tested, and the killer strain is immune to the effect of its own toxin. The effect of the killer toxin is dependent both on its own potency and the susceptibility of lawn cells under selected conditions. Since we found that high IAA concentrations inhibit the growth of yeasts. And we proposed that IAA secretion may be a crucial strategy for wild fungi to increase their competitiveness. Thus, we expect to see that the yeasts with high IAA producing ability should be more tolerant to IAA. In our data, a negative correlation was observed between IAA production and the inhibition ratio for IAA. This finding indicates the populations of high IAA-producing yeasts have evolved tolerance when exposed to this antagonistic compound. This observation supports our hypothesis that yeasts secrete IAA as an inhibiting agent to prevent the growth of competitors. We suggest that for each yeast species, an optimal concentration of IAA promotes growth, and that such effects are strain dependent. Although IAA produced by yeasts can inhibit the growth of specific strains competing for resources, in some cases, the produced IAA can stimulate the growth of other yeasts or their own growth. This finding implies that IAA may play a crucial role in determining interference competition among fungal species that occupy the same niche. Thus, IAA secretion may be a crucial strategy for wild fungi to increase their competitiveness.

In summary, we showed the variation in IAA production by wild *Saccharomyces* yeast strains from diverse ecological sources. Our data support the hypothesis that the inhibition of the growth of competitors by secreted IAA is an example of interference competition among yeast species. The profile of quantitative trait alleles has proven useful to reverse quantitative genetics approaches. It supports the use of systems genetics to synthesize the molecular basis of phenotypic variation across multiple strains. The results here certainly will be useful in the future to elucidate IAA biosynthesis pathways in yeast.

## Supporting Information

S1 Table IAA production by wild *Saccharomyces* yeasts in YPD medium supplemented with and without 0.1% L-tryptophan. Data are expressed as mean ± SD (μg/mL). Negative absorbance indicates no detectable IAA in the cultures. The detailed information of each strain is provided in Supplementary Tables 1 and 2 in the study of Liti et al. [23].
(DOCX)
