# Supplementary material for: Variation in Indole-3-Acetic Acid Production by Wild Saccharomyces cerevisiae and S. paradoxus Strains from Diverse Ecological Sources and Its Effect on Growth
Source: PLoS One. 2016 Aug 2;11(8):e0160524. doi: 10.1371/journal.pone.0160524 (PMC4970732; doi:10.1371/journal.pone.0160524)
Supplement: S1 Table — Data are expressed as mean ± SD (μg/mL). Negative absorbance indicates no detectable IAA in the cultures. The detailed information of each strain is provided in Supplementary Tables 1 and 2 in the study of Liti et al. [23]. (DOCX) [file pone.0160524.s001.docx]

**S1 Table. IAA production by wild *Saccharomyces* yeasts in YPD medium supplemented with and without 0.1% L-tryptophan.** Data are expressed as mean ± SD (µg/mL). Negative absorbance indicates no detectable IAA in the cultures. The detailed information of each strain is provided in Supplementary Tables 1 and 2 in the study of Liti et al. [23].

| **strains** | **YPD with Trp** | **YPD w/o Trp** | **Ecological sources** |
| --- | --- | --- | --- |
| ***Saccharomyces cerevisiae*** |  |  |  |
| UWOPS05-227.2 | 287.0(±16.6) | 100.24(±6.33) | *Trigona* spp (stingless bee), collected near Bertam palm  flower |
| UWOPS83-787.3 | 190.8(±26.8) | -8.8(±3.3) | Fruit, *Opuntia stricta* |
| YJM981 | 178.3(±3.8) | -37.1(±3.1) | vagina of patient suffering from vaginitis |
| Y55 | 208.7(±21.0) | -9.8(±4.9) | Grape |
| UWOPS05-217.3 | 251.9(±57.7) | 35.8(±3.3) | Nectar, Bertram palm |
| YJM975 | 131.6(±14.9) | -39.5(±4.6) | vagina of patient suffering from vaginitis |
| 273614N | 190.4(±47.0) | -24.1(±6.2) | Clinical isolate (Fecal) |
| S288c | 186.2(±11.8) | -13.4(±6.1) | Rotting fig |
| L-1528 | 182.7(±6.3) | -8.7(±2.0) | Fermentation from must Cabernet |
| Σ1278b | 121.8(±5.5) | -13.7(±4.0) | Unknown |
| YJM978 | 120.7(±3.7) | -31.3(±10.0) | vagina of patient suffering from vaginitis |
| Y12 | 173.9(±26.4) | -1.9(±2.3) | Palm wine strain |
| YPS606 | 163.1(±15.2) | -1.8(±3.6) | Bark of *Quercus rubra* |
| 322134S | 110.5(±5.0) | -15.7(±1.6) | Clinical isolate (Throat sputum) |
| NCYC110 | 104.7(±14.6) | -17.4(±4.2) | Ginger beer from *Zingiber* *officinale* |
| YPS128 | 104.2(±9.5) | -4.4(±1.1) | Soil beneath *Q*. *alba* |
| L-1374 | 99.2(±5.2) | -15.8(±0.9) | Fermentation from must País |
| DBVPG1373 | 98.1(±22.6) | -23.8(±3.8) | Soil |
| DBVPG6044 | 95.8(±14.2) | -33.5(±4.3) | Bili wine, from *Osbeckia grandiflora* |
| DBVPG6765 | 95.0(±25.5) | -18.9(±6.2) | Unknown |
| BC187 | 90.7(±2.8) | -14.2(±0.7) | Barrel fermentation |
| DBVPG1106 | 67.1(±1.2) | -11.6(±1.3) | Grapes |
| YS9 | 50.4(±7.3) | -21.4(±3.9) | Baker strain (Le Saffre yeast, commercial) |
| UWOPS87-2421 | 33.3(±1.9) | -24.9(±0.3) | Cladode, *Opuntia megacantha* |
| UWOPS03-461.4 | 31.9(±9.8) | -11.3(±14.5) | Nectar, Bertram palm |
| 378604X | 14.5(±2.5) | -22.9(±0.4) | Clinical isolate (Sputum) |
| Yllc17_E5 | -27.8(±0.7) | -36.8(±0.6) | Wine |
| W303 | -7.0(±6.3) | -40.4(±3.5) | Unknown |
| ***Saccharomyces paradoxus*** |  |  |  |
| CBS432 | 238.4(±76.2) | -25.6(±15.0) | Bark of *Quercus* spp |
| Q69.8 | 172.5(±14.5) | -6.4(±4.4) | Bark of *Quercus* spp |
| DBVPG6304 | 167.6(±12.2) | -35.6(±2.2) | *Drosophila pseudoobscura* |
| JLD222 | 43.0(±9.7) | -36.7(±3.5) | Unknown |
| N-17 | 39.5(±2.3) | -19.9(±6.0) | Exudate of *Q*. *robur* |
| Q32.3 | 119.1(±22.9) | -14.3(±7.2) | Bark of *Quercus* spp |
| KPN3828 | 118.1(±29.3) | -32.0(±5.1) | Bark of *Q*. *robur* |
| Q31.4 | 115.0(±13.2) | 52.1(±6.6) | Bark of *Quercus* spp |
| Q95.3 | 111.8(±34.1) | -13.5(±2.3) | Bark of *Quercus* spp |
| Z1 | 107.8(±6.1) | -1.9(±4.6) | Bark of *Quercus* spp |
| Q89.8 | 100.7(±3.7) | -3.5(±8.3) | Bark of *Quercus* spp |
| S36.7 | 99.7(±11.5) | -12.9(±3.5) | Bark of *Quercus* spp |
| KPN3829 | 95.5(±19.9) | -27.6(±4.8) | Bark of *Q*. *robur* |
| Z1.1 | 94.9(±36.4) | -37.2(±12.8) | Bark of *Quercus* spp |
| Q59.1 | 93.9(±2.8) | -15.3(±2.9) | Bark of *Quercus* spp |
| Y8.5 | 90.3(±18.1) | -12.5(±2.3) | Bark of *Quercus* spp |
| T21.4 | 89.9(±11.6) | -11.9(±6.8) | Bark of *Quercus* spp |
| Q62.5 | 89.0(±10.7) | 10.3(±15.0) | Bark of *Quercus* spp |
| Q74.4 | 87.4(±2.8) | -13.8(±7.7) | Bark of *Quercus* spp |
| DBVPG4650 | 87.0(±36.5) | -26.7(±22.2) | Fossilized guano in a cavern |
| UWOPS91-917.1 | 76.0(±3.7) | -10.0(±7.8) | Flux of *Myoporum sandwichense* |
| W7 | 75.3(±35.4) | -14.9(±1.8) | Bark of *Quercus* spp |
| Y9.6 | 73.9(±24.1) | -16.5(±8.9) | Bark of *Quercus* spp |
| A4 | 69.8(±49.8) | -25.5(±20.0) | Bark of *Q*. *rubra* |
| Y7 | 68.1(±7.7) | -5.1(±4.3) | Bark of *Quercus* spp |
| CBS5829 | 62.0(±28.6) | -38.7(±15.9) | Mor soil, pH3.6 |
| YPS138 | 51.3(±11.1) | -3.1(±8.2) | Soil beneath *Q*. *velutina* |
| UFRJ50816 | 40.2(±8.8) | -42.5(±2.5) | *Drosophila* spp |
| N-43 | 37.6(±8.8) | -17.1(±9.0) | Exudate of *Q*. *mongolica* |
| Y6.5 | 32.2(±1.9) | -22.3(±7.4) | Bark of *Quercus* spp |
| IFO1804 | 32.1(±2.8) | -3.0(±2.3) | Bark of *Quercus* spp |
| N-44 | 26.6(±13.4) | 0.1(±2.6) | Exudate of *Q*. *mongolica* |
| Y8.1 | 26.1(±4.3) | -10.2(±11.6) | Bark of *Quercus* spp |
| A12 | 17.2(±13.1) | -9.2(±7.9) | Soil beneath *Q*. *rubra* |
| N-45 | 11.3(±1.0) | -2.0(±19.2) | Exudate of *Q*. *mongolica* |
| UFRJ50791 | -14.3(±21.0) | -34.7(±2.5) | *Drosophila* spp |
